# The technique for retrieving a fully migrated pancreatic duct stent using a snare placed over the proximal end of biopsy forceps

**DOI:** 10.1055/a-2573-7598

**Published:** 2025-04-15

**Authors:** Kazuya Sumi, Naoya Patrick Terai, Hisaki Kato, Yuki Kawasaki, Jun Ushio, Takayoshi Ito, Haruhiro Inoue

**Affiliations:** 1 378609 Digestive Diseases Center, Showa University Koto-Toyosu Hospital, Tokyo, Japan

Pancreatic stents (PSs) are predominantly used to treat pancreatitis and pancreatic duct (PD) stenosis. However, stent migration is occasional. Several methods have been reported for migrated PS retrieval 1 2 3 4 5 , but removal remains challenging, as excessive manipulation increases the risk of duct injury and pancreatitis.

A 70-year-old woman with acute pancreatitis developed distal PD stenosis and a pseudocyst, for which a PS (7 Fr × 12 cm) was inserted ( Fig. 1 ). PS insertion resulted in pseudocyst size reduction ( Fig. 2 ). After 3 months, endoscopic retrograde cholangiopancreatography was performed to examine the stenosis. During the procedure, the PS was grasped with rat-tooth forceps but adhered firmly at the stenotic site. Attempted traction caused damage and migration completely into the PD ( Fig. 3 ). Standard forceps could not be inserted because of mild PD dilation. Furthermore, retrieval using a balloon catheter was unsuccessful after multiple attempts.

**Fig. 1:**
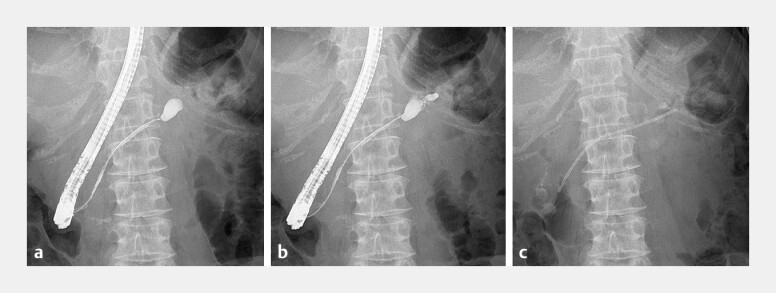
**a** Pancreatography revealing main PD stenosis. **b** Pancreatography revealing a pseudocyst that communicates with the duct immediately distal to the stenosis. ( **c** ) A PD stent was inserted to distally extend, reaching beyond the stenosis. PD, pancreatic duct.

**Fig. 2:**
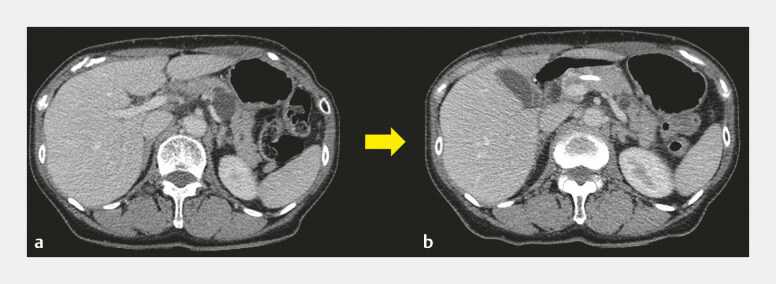
**a** CT before the PD stent insertion. **b** One month after the PD stent insertion, and CT demonstrated a significant pseudocyst size reduction.

**Fig. 3:**
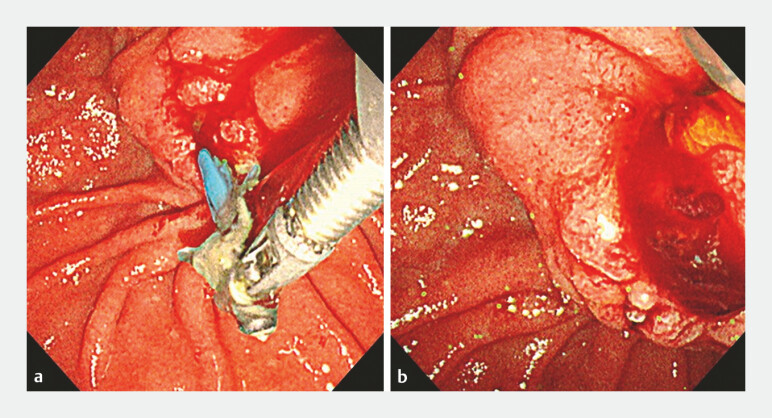
**a** Traction was applied to the proximal end of the stent with rat-tooth forceps. **b** The stent was firmly adhered at the stenotic site and damaged, becoming migrated into the PD.

Fluoroscopic guidance allowed small-caliber biopsy forceps (BF) to grasp the damaged portion of the stent; however, extraction was not possible. Strong traction resulted in a loss of grasp. The damaged portion was grasped again with BF, but repeated attempts posed an increased risk of pancreatitis without guaranteeing success. BF provided excellent maneuverability, but its grasping force was limited. To overcome this challenge, we developed a snare-assisted retrieval method using a snare while maintaining a BF grasp. In this method, a snare (thin-diameter crescent snare, Olympus) was guided through the proximal end of the forceps to the stent, securing it while maintaining the BF hold. The stent was successfully retrieved without complications ( Fig. 4 ). Postprocedure pancreatography revealed no significant PD injury ( Video 1 ).

**Fig. 4:**
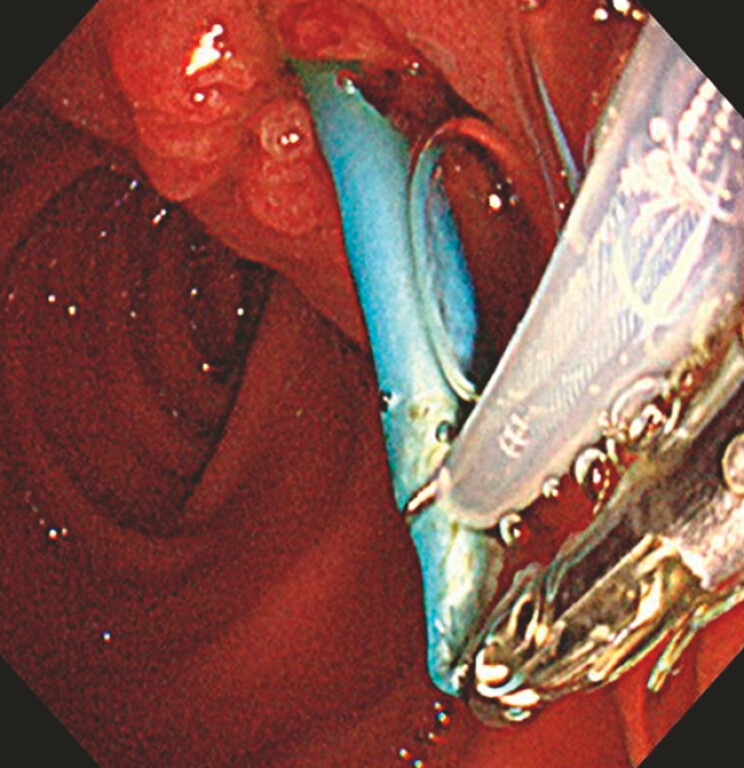
The damaged part of the stent was grasped and was successfully retrieved with traction from both devices.

Retrieval of a fractured and migrated pancreatic duct stent using a snare that passed through the proximal end of biopsy forceps was achieved by coordinated force from both devices for simple and safe extraction.Video 1

When BF traction alone is insufficient, the snare-assisted technique enables direct endoscopic visualization, thereby minimizing unnecessary manipulation within the PD. This approach represents a simple, safe, and effective method of stent retrieval without compromising BF maneuverability.

Endoscopy_UCTN_Code_TTT_1AR_2AI

## Notes

Endoscopy E-Videos https://eref.thieme.de/e-videos
*E-Videos* is an open access online section of the journal *Endoscopy* , reporting on interesting cases and new techniques in gastroenterological endoscopy. All papers include a high-quality video and are published with a Creative Commons CC-BY license. Endoscopy E-Videos qualify for HINARI discounts and waivers and eligibility is automatically checked during the submission process. We grant 100% waivers to articles whose corresponding authors are based in Group A countries and 50% waivers to those who are based in Group B countries as classified by Research4Life (see: https://www.research4life.org/access/eligibility/ ). This section has its own submission website at https://mc.manuscriptcentral.com/e-videos .
